# A gain-of-function mutation in *head involution defective* , *Wrinkled,* causes precocious cell death of wing epidermal cells in *Drosophila*

**DOI:** 10.17912/micropub.biology.000703

**Published:** 2022-12-20

**Authors:** Takumi Ohta, Teiichi Tanimura, Ken-ichi Kimura

**Affiliations:** 1 Laboratory of Biology, Hokkaido University of Education, Sapporo Campus, Sapporo, Japan; 2 Department of Biology, Graduate School of Science, Kyushu University, Fukuoka, Japan; 3 Present address: Division of Biological Science, Graduate School of Science, Nagoya University, Nagoya, Japan.

## Abstract

In *Drosophila* , wing epidermal cells undergo programmed cell death as the last step of metamorphosis. The aim of this study was to evaluate the role of *hid* , particularly the *Wrinkled* mutation ( *hid ^W^* ), an allele of *hid* , in the cell death. The wing epithelial cell death is suppressed by loss-of-function mutation of *hid* , indicating that the death is governed by a cascade involving *hid* . Examination of the cell death in *hid ^W^* showed that precocious death started at G stage, 3 h before eclosion. Thus, mutated-HID in the *hid ^W^* mutant was activated at G stage, supporting the gain-of-function effect of *hid ^W^* mutation.

**Figure 1. A proapoptotic gene, hid, is involved in wing epidermal cell death after eclosion:**
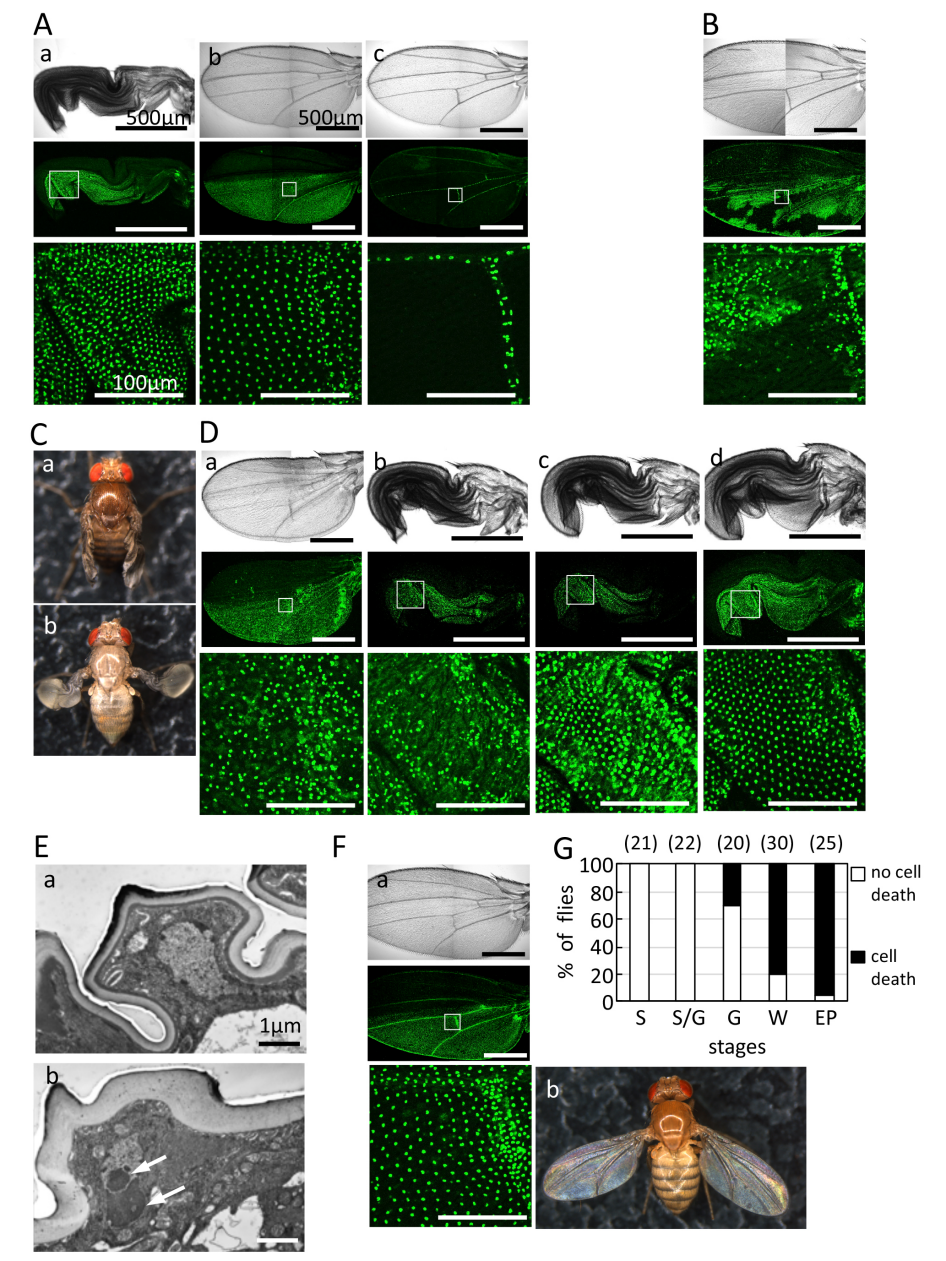
A. Wing epidermal cells marked with nuclear-localized GFP in *en-Gal4 UAS-GFPN* flies at eclosion (a), just after wing expansion (b), and at 1 h after eclosion (c). The wing epidermal cells are present till 0 h after wing expansion, but disappear at 2 h after eclosion because of PCD. Nuclear-localized GFP is detectable only in the nuclei of vein cells (c). B. Even at 2 h after eclosion, wing epidermal cells in the *hid* mutant of *UAS-GFPN en-Gal4/+; th hid ^A206^ ri/mwh H99 ri* were still present because PCD was suppressed. C. Wing phenotypes in a *Wrinkled (hid ^W^ )/TM6b* mutant at a few days after eclosion (a). The wings show wrinkled and unexpanded phenotypes. The *hid ^W^* heterozygous mutant just after wing expansion (b). The wings show balloon and blistered phenotypes. After reabsorption of the body fluid, the wings shrunk to the size of those in (a). D. Wing epidermal cells in the heterozygous mutant, *UAS-GFPN en-Gal4/CyO; hid ^W^ /TM6b* just after wing expansion (a) and at EP (b), G (c) and S (d) stages. The *Wrinkled* ( *hid ^W^* ) mutation causes precocious cell death of wing epidermal cells after G stage. E. Transmission electron microscopy in a wing epidermal cell at 0 h after eclosion in CS (wild type) (a) and *hid ^W^ /TM6b* mutant (b). The arrow in (b) indicates condensed chromatin structure. Suppression of blistered wing phenotype (g) and precocious cell death (H) of *hid ^W^* mutation by ectopic expression of anti-apoptotic protein p35 in *en-Gal4 UAS-GFPN/+; UAS-p35/hid ^W^* . (i–k) The nuclei marked with GFPN in *UAS-GFPN en-Gal4/CyO; hid ^W^ /TM6b* flies were detected in allmost cells at S stage (i), but decreased in G stage (j) and EP stage (k). F. Suppression of and precocious cell death (a) and blistered wing phenotype (b) of *hid ^W^* mutation by ectopic expression of anti-apoptotic protein p35 in *en-Gal4 UAS-GFPN/+; UAS-p35/hid ^W^* . G. Appearance of precocious cell death at various stages of pharate adult in *UAS-GFPN en-Gal4/CyO; hid ^W^ /TM6b* . Black bars and white bars indicate percentage of flies showing cell death and not showing cell death in epidermal cells, respectively. Values in parentheses indicate the number of flies examined. The white boxed regions in middle panels of A (a-c), B, D (a-d) and F are enlarged in panels below at higher magnification. Scale bars: 500 and 100 µm in top/middle panels and bottom panels, respectively, of A (a-c), B, D (a-d) and F. 1 µm in E (a, b).

## Description

Programmed cell death (PCD) is important for eliminating unnecessary or deleterious cells during the building of the mature body in developmental processes (Jacobson *et al* ., 1997, Vaux and Korsmeyer, 1999). Most physiological cell death appears to be induced via common core effectors, including caspase proteases that are central components of the machinery responsible for apoptosis. In *Drosophila melanogaster* , four pro-apoptotic genes, *reaper* ( *rpr* ) (White *et al* ., 1994), *head involution defective* ( *hid* ) (Grether *et al* ., 1995), *grim* (Chen *et al* ., 1996), and *sickle* ( *skl* ) (Christich *et al* ., 2002; Srinivasula *et al* ., 2002; Wing *et al* ., 2002a), induce cell death. The products of these cell death genes have the potential to bind to inhibitory apoptotic protein (IAP) to prevent its function (Hay *et al* ., 1995, Vucic *et al* ., 1997, 1998, Wang *et al* ., 1999, Goyal *et al* ., 2000). Free IAP protein can bind to and activate the initiator caspase (Kaiser *et al* ., 1998, Hawkins *et al* ., 1999, Meier *et al* ., 2000). Thus, the products of these pro-apoptotic genes activate the caspase, resulting in the induction of cell death. Although these pro-apoptotic genes act through a similar mechanism, various signals converge onto pro-apoptotic genes via a different signaling systems in different tissues during development. How these extracellular signals and signal transduction pathways are linked with the apoptotic machinery remains unclear.

After wing extension, cell death is initiated in wing epidermal cells in *Drosophila* (Johnson and Milner, 1987, Kimura *et al* ., 2004, Link *et al* ., 2007). The death of wing epidermal cells involves the breakdown of nuclear membranes, followed by the fragmentation of nuclear DNA. Transmission microscopy revealed that cell death occurs through autophagy-like processes. Ectopic expression of an anti-apoptotic gene, p35, inhibited cell death, indicating the involvement of caspases (Kimura *et al* ., 2004). The knockout of apical caspases of the apoptosome components *dark* and *dronc* or effector caspase *drice* in the wing epidermal cells suppressed cell death (Xu *et al* ., 2005, Link *et al* ., 2007). Although death initiates after wing extension, the death competence of wing epidermis is acquired 3 h before eclosion (Kimura *et al* ., 2004). Garcia-Hughes *et al* . (2015) showed that transcription of *hid* and accumulation of HID protein is induced prior to cell death in the wing epidermal cells, suggesting that the pro-apoptotic gene, *hid* , is regulated post-translationally during the wing epidermal cell death. In this study, the role of *Wrinkled* ( *hid ^W^* ), the dominant allele of *hid* (Abbot and Lengyel, 1991) in cell death was examined.

Wings of the newly eclosed wild-type flies are folded (Fig. 1Aa) but expand within 30 min following a certain set of behaviors (Fig. 1Ab, Movie 1, Baker and Truman, 2002). A strain of flies carrying *en-Gal4* *UAS-GFPN* expressed nuclear-localized GFP in epidermal cells in the posterior compartment of the wings (Fig. 1Aa,b). After wing expansion, GFP expression disappeared in the wing epidermal cells within 2 h after eclosion (Fig. 1Ac), indicating that PCD of the wing epidermal cells had occurred (Kimura *et al* ., 2004).

To confirm the involvement of a pro-apoptotic gene, *hid* , in the wing epidermal cells we examined the effects of loss-of-function mutations in the pro-apoptotic gene *hid* . In the *hid* mutant of *+* / *UAS-GFPN en-Gal4; th hid ^A206^ ri* / *mwh H99 ri* , many cells expressing GFP were observed even at 2 h after eclosion (Fig. 1B). This indicates that wing epidermal cell death is mediated by HID. These results are consistent with those of a previous report (Garcia-Hughes *et al* ., 2015).

*Wrinkled* ( *hid ^W^* ), a dominant allele of *hid* , is a mutation resulting in an unexpanded wing phenotype (Fig. 1Ca; Abott and Lengyel, 1991). We examined the wing extension behavior after eclosion in *hid ^W^ /TM6b* mutants (Movie 2). Although these mutants showed normal wing extension behavior, adhesion of two layers of the dorsal and ventral cuticles of the wings was disrupted, resulting in the formation of blister wings during extension (Fig. 1Cb). After absorption of the hemolymph, the wings became crumpled (Fig. 1Ca).

Previously, we reported that cell death in wing epidermal cells plays an important role in wing maturation, and precocious cell death results in blistered wing (Kimura *et al* ., 2004). To examine whether precocious cell death caused blistered wing in the *hid ^W^* mutant, we evaluated the fate of wing epidermal cells in *en-Gal4 UAS-GFPN/CyO; hid ^W^ /TM6b* flies. In the *hid ^W^* mutants, cell death had proceeded even just after wing spreading (Fig. 1Da). Nuclei marked by GFP were not observed in some regions of the wings, and cells expressing GFP in the cytoplasm were scattered. Morphological studies using transmission electron microscopy revealed that the wing epidermal cell in the wild-type fly showed a healthy spherical nucleus with some electron dense materials at eclosion (Fig. 1Ea). However, some cells in the *hid ^W^* mutant possessed condensed chromatin, typical dying feature, even at eclosion (Fig. 1Eb). These results indicate that *hid ^W^* is a gain-of-function mutation that causes precocious cell death in wing epidermal cells.

PCD is regulated by promoting or inhibiting caspase activation in many cases (Meier *et al* ., 2000). To determine whether caspase dominates cell death in *hid ^W^* mutants, we examined the effect of forced expression of the anti-apoptotic protein baculovirus p35. p35 can act as a specific caspase inhibitor (Zhou *et al* ., 1997). We followed the fate of wing epidermal cells in *en-Gal4 UAS-GFPN/+; UAS-p35/hid ^W^* adults. During wing extension, when the cells died in *hid ^W^* mutants, GFP was observed in the nuclei (Fig. 1Fa). Thus, precocious cell death by *hid ^W^* was inhibited by p35, indicating that the cell death is caspase-dependent. The flies recovered from the balloon wing phenotype during wing expansion and showed a flat expanding wing (Fig. 1Fb).

In the wild-type flies of *en-Gal4* *UAS-GFPN,* no wing epidermal cell death is seen until at wing expansion after eclosion (Kimura *et al* ., 2004). Then, we investigated the time at which precocious cell death began in the *hid ^W^* mutant (Fig. 1Db-d, G). At S stage in pharate adults (Fig. 1Dd), GFP-expressing nuclei were observed throughout the region, indicating that the cells had not died. From G stage or later, breakdown of the nuclear membrane was observed in many cells, indicating that precocious cell death was induced (Fig. 1Db, c, G). Thus, altered-HID in the *hid ^W^* mutant should be activated even at G stage.

Wing epidermal cell death occurs after wing expansion. Studies using neck-ligation and hemolymph injection confirmed that a hormonal factor directly triggers cell death (Kimura *et al* ., 2004). This hormone would be bursicon, as bursicon silencing in the central nervous system generated cell death-defective phenotypes (Garcia-Hughes *et al* ., 2015). The hormonal signal is transmitted via the G-protein-coupled receptor of the rickets and cAMP/PKA signaling pathway (Kimura *et al* ., 2004). After being triggered by a hormonal signal, the death of wing epidermal cells proceeds within 1 h. Rapid induction of cell death is controlled by post-translational, rather than by transcriptional regulation. Analyses of the loss-of-function of the *hid* mutant showed that *hid* is involved in wing epidermal cell death. However, wing epidermal cell death does not occur until the death-triggering hormone is released after eclosion, even though wing epidermal cells already expressed *hid* mRNA and HID protein (Garcia-Hughes *et al* ., 2015). The HID amino acid sequence contains three consensus PKA phosphorylation sites (Grether *et al* ., 1995), suggesting that HID is a direct target of PKA. Phosphorylation of HID by PKA may activate HID.

In the *hid ^W^* mutant, precocious cell death was induced at approximately G stage and later, which was similar in timing to the competence of death induced by cAMP/PKA activation (Kimura *et al* ., 2004). Thus, the product of mutated *hid ^W^* appears to be active without hormonal signals, indicating that the mutant HID ^W^ is constitutively active. Investigations on the PKA phosphorylation site in HID and the molecular nature of the gain-of-function mutation *hid ^W^* would provide insight into these mechanisms.

## Methods

**Fly strains**

Flies were raised on cornmeal-yeast medium at 25°C under constant illumination. Canton-S (CS) flies were used as the wild-type strain. To follow the fate of epidermal cells after eclosion, wing epidermal cells were marked with nuclear-localized GFP (GFPN). A strain carrying both *engrailed-Gal4* ( *en-Gal4* ) (Dormand and Brand, 1998) and *UAS-GFPN* (Shiga *et al* ., 1996) on the same second chromosome was used as the wild-type. The *en-Gal4* line was used to express the following transgenes: *UAS-p35* (anti-apoptotic baculovirus protein; Hey *et al.* , 1995, Zhou *et al* ., 1997) using the GAL4/UAS expression system (Brand and Perrimon 1993).

A line of *Wrinkled* ( *hid ^W^* ) mutation, a dominant allele of *hid* , was obtained from the Kyoto stock center. A strain carrying the loss-of-function mutation of *hid* ; *th hid ^A206^ ri/TM6b* and *mwh* *Df (3L) H99 ri* /TM6b (Abbot and Lengyel, 1991, Grether *et al* ., 1995) were a gift from H. Steller.

**Observation of wing epidermal cells and detection of cell death**

The wings were dissected in phosphate-buffered saline (PBS) and mounted with PBS on a slide glass. Images of expression of GFP in the posterior compartment of wing blade were obtained with the Leica (Wetzlar, Germany) confocal microscope TCS SPE using LAS AF software. The disappearance of GFPN beneath the wing hairs (each cell in wing blade produces a single wing hair) was considered to indicate cell death. Pupal stages before eclosion were classified at S, S/G, G, W, and EP under a dissection microscope in accordance to the classification provided by Kimura and Truman (1990); those stages are at about 9 hr, 6 hr, 3 hr, 50 min, and 40min before eclosion, respectively.

**Histology**

The wings were fixed for electron microscopy (EM) in 2.5% glutaraldehyde in 0.1 M phosphate buffer and embedded in Epon 812 using standard procedures. EM sections were stained with uranyl acetate and lead citrate and examined with a JEOL 1010 electron microscope (Tokyo, Japan).

## Reagents

y ^1^ w*; P{en2.4-GAL4}e16E (BDSC, 30564)

*w ^1118^ ; P{w ^+mC^ =UAS-GFP.nls}14* (DGGR, 107870)

*Wrinkled* ( *hid ^1^ , hid ^W^* ) (DGGR, 106018)

*th hid ^A206^ ri/TM6b* (Abbot and Lengyel, 1991)

*mwh* *Df (3L) H99 ri* /TM6b (Abbot and Lengyel, 1991)

w*;; P{UAS-p35.H}BH2 (BDSC, 5073)

## Extended Data

Description: Movie 1 Wing spreading after eclosion in a wild-type fly A newly eclosed wild-type fly (CS strain) with folded wings walked around, and then expanded it wings within 30 min after eclosion.. Resource Type: Audiovisual. DOI: 10.22002/ybfw7-k4s15

Description: Movie 2 Blister wings caused by precocious cell death in a hidW mutant Although a hidW /TM6b mutant fly showed normal wing extension behavior, adhesion of two layers of the dorsal and ventral cuticles of the wings was disrupted by precocious cell death, resulting in the formation of blister wings during extension.. Resource Type: Audiovisual. DOI: 10.22002/x06ax-19190
